# Characterizing chapeau de gendarme in pediatric epilepsy through systematic video‐EEG evaluation

**DOI:** 10.1002/epi.70396

**Published:** 2026-07-23

**Authors:** Hanna Barbara Brinkmann, Lea Nakamura, Jan Schönberger, Julia Jacobs, Victoria San Antonio‐Arce, Kerstin Alexandra Klotz

**Affiliations:** ^1^ Department of Neuropediatrics and Muscle Disorders, Center for Pediatrics, Medical Center, Faculty of Medicine University of Freiburg Freiburg im Breisgau Germany; ^2^ Epilepsy Center, Medical Center, Member of the European Reference Network for Rare and Complex Epilepsies EpiCARE, Faculty of Medicine University of Freiburg University of Freiburg Freiburg im Breisgau Germany; ^3^ Hotchkiss Brain Institute and Alberta Children's Hospital Research Institute University of Calgary Calgary Alberta Canada; ^4^ Department of Neuropediatrics, Member of the European Reference Network for Rare and Complex Epilepsies EpiCARE, University Hospital Bonn University Bonn Bonn Germany

**Keywords:** focal epilepsy, hyperkinetic, ictal pouting, semiology

## Abstract

**Objective:**

Chapeau de gendarme (CdG) is a subtle but clinically relevant semiology that has been scarcely studied in children. Previous studies have primarily focused on its localizing value in small, surgical cohorts. This study aimed to systematically characterize frequency and clinical features of CdG across the pediatric age spectrum.

**Methods:**

We retrospectively analyzed video‐electroencephalography (EEG) monitoring of 264 children with epilepsy (0–17 years). Up to 10 seizures per type were reviewed per patient. CdG was defined as a symmetrical downward contraction of both mouth corners and chin contraction lasting ≥5 s. CdG characteristics, co‐occurring semiologies, and EEG patterns were assessed and compared to the cohort without CdG.

**Results:**

CdG was identified in 28 of 264 patients (10.6%), corresponding to 73 seizures. The median age at first observed CdG was 12.3 years (range 1.3–16.8), with a trend toward increasing frequency in older children. All but one case occurred in focal epilepsies, which were significantly more frequent in the CdG cohort than in those without CdG (*p* = .009). CdG appeared early in the seizure course (≤5 s in 61.6%) and more often during sleep (*p* < .001). Hyperkinetic behavior occurred significantly more often in CdG seizures (*p* < .001). Progression to bilateral tonic–clonic seizures was recorded in 20.5% of CdG seizures (17.9% of patients with CdG) and occurred more frequently in the CdG cohort than in patients without CdG (*p* < .001). The first ictal scalp EEG pattern most frequently involved the frontal lobe (57.7%).

**Significance:**

CdG occurred in a notable proportion of pediatric patients across the entire age range, with a descriptive tendency toward higher occurrence in older children. These findings provide an essential foundation for future investigations exploring developmental and network mechanisms underlying this semiology.

**Key points**

- Chapeau de gendarme (CdG) occurred in 10.6% of pediatric patients from infancy to adolescence, with a tendency to higher frequency in older children.
- CdG was typically an early seizure sign, emerging within the first seconds of seizure onset.
- CdG frequently co‐occurred with hyperkinetic behavior and in seizures progressing to bilateral tonic–clonic seizures.
- The first ictal scalp electroencephalography (EEG) patterns were most often seen frontal, followed by temporal; no clear lateralization was observed.

## 1 INTRODUCTION

Epilepsy is one of the most common neurological disorders in children.^1^ Epileptic seizures manifest clinically through their semiology. Careful recognition and description of semiology are essential, as they contribute to seizure classification, localization of the epileptogenic zone, assessment of underlying networks, and ultimately to the choice of therapy.^2^ In children, semiology differs from that in adults. Non‐motor features may be difficult to detect in younger patients due to limited language and self‐reporting skills.^3^ Moreover, age‐dependent brain maturation influences the expression and evolution of seizure semiologies: elementary signs predominate in early childhood, whereas complex patterns, such as hyperkinetic behavior (HKB) and bilateral tonic–clonic seizures (BTCS), become more common with increasing age.^3^, ^4^ Of note, the localizing and lateralizing value of seizure semiology also changes with age.^5^ Certain semiologies, particularly those appearing early after seizure onset, may help localize the epileptogenic zone.^6^ In infants and toddlers, however, ictal signs are often bilateral or poorly differentiated, limiting their localizing value. With advancing brain maturation, semiologies become more specific and provide increasingly reliable localizing and lateralizing information.^7^

One subtle and little‐studied semiology is chapeau de gendarme (CdG), a symmetrical downward contraction of both mouth corners with chin contractions lasting several seconds, resembling the shape of the French gendarme's hat under Napoleon.^8^, ^9^ It has been mainly described in surgical cohorts focusing on the localizing value of CdG; broader studies in non‐surgical cohorts do not exist. In adults, CdG is typically an early seizure sign, often co‐occurring with HKB, tonic posturing, or vegetative symptoms.^9^ According to a recent systematic review by Moser et al.,^10^ CdG has so far been observed exclusively in focal epilepsies. The predominantly reported etiologies were structural, most often focal cortical dysplasia^9^, ^10^, ^11^; autoimmune encephalitis has been reported in only a single case.^12^ Seizures with CdG are associated with different regions including frontal, temporal, and insular areas.^9^, ^10^, ^11^, ^13^ No lateralizing value has been demonstrated.^10^ In children and adolescents, evidence is even more limited to single case reports and small series, also focusing primarily on localizing value in surgical cohorts. Reported pediatric cases share several features with adults: early occurrence, lack of lateralizing value, and predominantly structural etiologies, especially focal cortical dysplasia, although one tumor has also been reported.^11^, ^14^, ^15^, ^16^ CdG may occur together with HKB, mainly in older adolescents.^9^, ^14^ The youngest published patient with CdG was 14‐months‐old,^16^ but most reported cases involved children and adolescents 6–18 years of age. However, the focus on surgical patients with stereo–electroencephalography (EEG) likely introduces reporting bias and underrepresents younger children. Small sample sizes and skewed age further limit conclusions on the frequency of CdG in childhood epilepsy and its semiological evolution across age groups.

The aim of this study was therefore to investigate CdG in a large pediatric cohort, spanning the full age range and independent of etiology and EEG modality. Unlike previous studies that focused primarily on the localizing value of CdG in highly selected surgical populations, our objective was to systematically characterize its frequency and clinical features in children.

## 2 METHODS

### 2.1 Patient selection and clinical data

This retrospective study was conducted at the Epilepsy Center of the University Hospital Freiburg. We included video‐EEG monitoring (VEM) of children and adolescents with epilepsy ages 0–17 years recorded between January 1, 2013 and December 31, 2019. If multiple VEMs were performed, all recordings more than 12 months apart were included. For VEMs performed within 12 months, only the first was considered, unless subsequent recordings contained any new seizure types. VEMs were excluded if they did not capture any seizures; contained only unclear, questionable, or purely subclinical seizures; or if the recording could not be evaluated, for example, patient outside the camera field, video quality too poor, and/or video not accessible. The following clinical data were collected: age at VEM, epilepsy classification and etiology, age at first seizure, seizure frequency, anti‐seizure medication, and epilepsy surgery (type and outcome, if performed).

### 2.2 Video analysis

All videos were independently analyzed by two raters, one of whom was a certified epileptologist (Rater 2). For every patient, up to 10 seizures per type were analyzed. If fewer than 10 seizures per type were recorded or assessable, all available seizures were evaluated. For each seizure, the first two semiologies identifiable on video or EEG (e.g., heart rate increase) were documented. In cases where the clinical seizure onset was unclear, the EEG onset was used, and the first subsequent clinical sign was defined as the initial clinical manifestation. A descriptive approach was used when reporting the sequence and co‐occurrence of semiological signs. For seizure classification, however, the first prominent sign according to the 2017 seizure classification was applied.^17^

Particular emphasis was placed on the identification of CdG and HKB. For this purpose, each seizure was reviewed for a maximum of 10 min (or until its termination if shorter). When present, the onset timing of CdG or HKB was documented, as well as the duration of CdG. In addition, the occurrence of BTCS was recorded. If a semiological feature could neither be confirmed nor ruled out (e.g., due to body position or incomplete camera view), this was also noted. Cases for which the two raters did not reach concordant assessments were jointly reviewed, and seizures without consensus were excluded from further analysis. Vigilance at seizure onset was determined from EEG when possible or from video (movement, heart rate, artifacts, eye closure) otherwise, and classified as indeterminable if still unclear.

### 2.3 Criteria for classification of CdG and HKB

CdG was defined as a symmetrical downward contraction of both mouth corners with accompanying chin contractions, persisting for at least 5 s. Continuous visibility of the mouth area (both corners) during this period was required (Figure 1).

**FIGURE 1:**
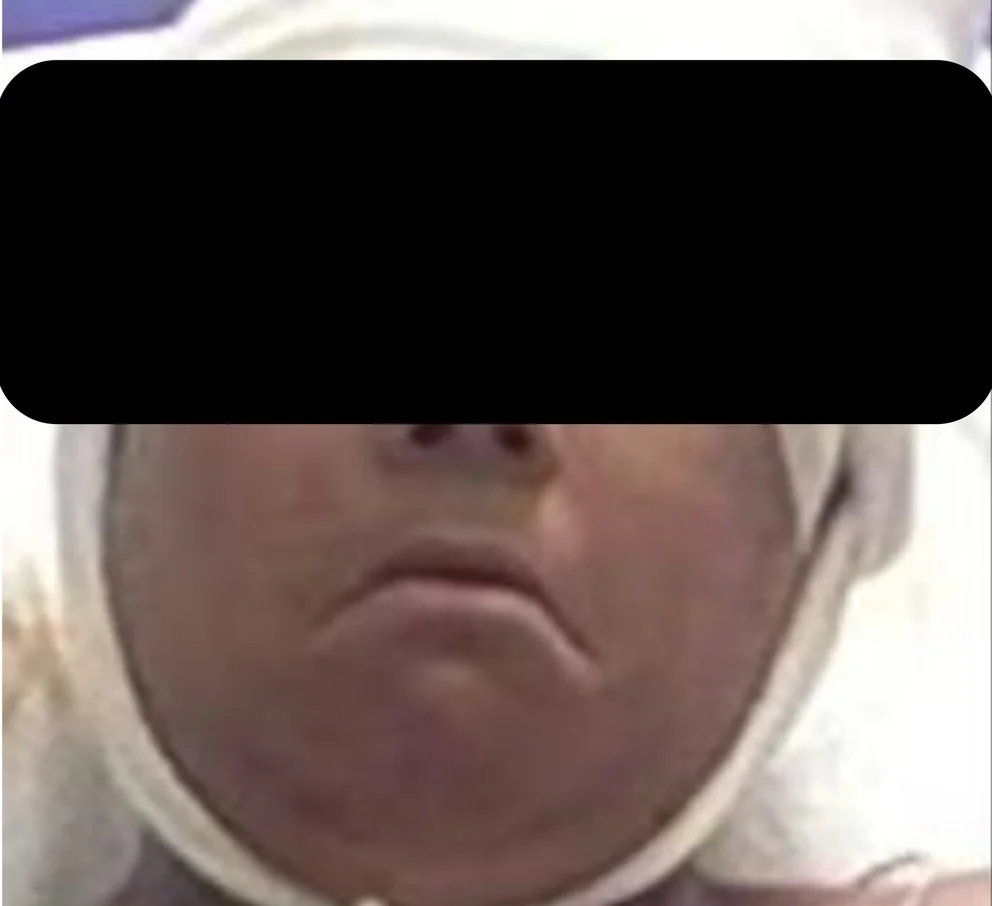
Chapeau de gendarme (CdG) in a 14.1‐year‐old patient (Patient 18, Table S1; focal cortical dysplasia type IIa of the left anterior cingulate cortex). Symmetrical contraction of the lip corners with associated chin contraction is shown. A fearful or startled facial expression was observed during the seizure. Due to privacy restrictions, the upper part of the face is obscured; therefore, the overall facial expression can only be partially appreciated.

HKB was defined as strong, rapid, sweeping, and situationally inappropriate complex movements of the trunk and/or the proximal extremities (e.g., kicking, boxing, body rocking).^8^, ^18^

### 2.4 Ictal EEG analysis

In seizures with CdG, the first identifiable ictal EEG pattern was determined by Rater 2 using the EEG software *Profusion* (Compumedics Limited, Abbotsford Victoria, Australia). The occurrence of the ictal pattern was assigned to the different lobes (frontal, temporal, parietal, occipital, insular), classified as generalized or, if undeterminable, labeled as unknown (diffuse and bilateral pattern, or only lateralization possible). If initial seizure patterns on scalp EEG involved more than one lobe (e.g., fronto‐temporal), both regions were considered in the evaluation. A few EEG recordings were obtained from intracranial electrodes. Patterns from these recordings were evaluated, taking into account whether the involved region was regarded as the true seizure‐onset zone at final assessment, resection was performed, and postoperative seizure freedom was achieved.

### 2.5 Statistical analysis

Statistical analyses were performed using *GraphPad Prism* (version 10.2.3; GraphPad Software, Inc., California, USA). The significance level was set at *α* < .05. Descriptive statistics are presented as mean ± standard deviation (SD) and/or median and range, and as percentages where applicable. Normality was tested with the D'Agostino and Pearson test. As the data were not normally distributed, the Mann–Whitney *U* test was applied to comparisons of continuous variables between groups. Group comparisons of categorical variables (age group distribution, occurrence of HKB and BTCS) were performed using Fisher's exact test. Spearman's rank correlation was used to assess associations between age and CdG occurrence.

## 3 RESULTS

### 3.1 Study flow and population

During the study period, a total of 683 VEMs were performed in children and adolescents. Of these, 366 VEMs were excluded for one or more of the following reasons: patient age older than 18 years, absence of epilepsy, lack of recorded clinical seizures events, limited assessment of the VEM (e.g., patient outside the camera field, poor video quality, or unavailable video recordings), or when multiple VEMs were performed within 12 months without any new seizure types. From the remaining 317 VEMs with a total of 2906 recorded seizures, 158 events were excluded because they were not clearly epileptic, lacked discernible clinical signs, could not be distinguished within seizure clusters, or for technical reasons. Ultimately, 2748 seizures from 317 VEMs in 264 pediatric patients were included in the analysis. The number of VEMs exceeded the number of patients, as several children underwent two (14%), three (1.1%), or more than three recordings (1.1%). For the entire cohort, the mean age (SD) at the first assessable VEM was 9 years (4.9) and 9.2 years across all VEMs (4.9). The age distribution of the entire study cohort was as follows: 0 to <3 years 12.9%, 3 to <6 years 17%, 6 to <9 years 18.6%, 9 to <12 years 15.5%, 12 to <15 years 22.7%, and 15 to <18 years 13.3%.

### 3.2 Characteristics of patients with CdG

Among the analyzed patients, CdG was observed in 10.6% (*n* = 28) of all patients, corresponding to 30 VEMs (28 scalp EEG recordings, 2 intracranial EEG recordings) and 73 seizures. Within the CdG cohort, all epilepsies were classified as focal, except for one case with an undetermined focal or generalized onset. Compared to the non‐CdG cohort (focal *n* = 202 [85.6%], generalized *n* = 20 [8.5%], unclassified *n* = 14 [5.9%]), focal epilepsy was significantly more frequent in patients with CdG (*p* = .009). The predominant etiology in the CdG cohort was structural, most frequently focal cortical dysplasia (17.9%). Eight patients underwent epilepsy surgery, with Engel class I outcome in six, Engel class II in one, and missing outcome data in one patient (Table S1). Additional clinical characteristics of patients with CdG are summarized in Table 1.

**TABLE 1:** Characteristics of patients with CdG (*n* = 28).

Characteristic	Data
Female sex, *n* (%)	15 (53.6)
Age at first CdG, years; median [range]	12.3 [1.3–16.8]
Age at epilepsy diagnosis, years; median [range]	4.9 [0.1–14]
Etiology, *n* (%)	
Structural	11 (39.3)
Focal cortical dysplasia	7 (25)
Tumor	4 (14.3)
Structural‐Genetic	4 (14.3)
Tuberous sclerosis	2 (7.1)
Aicardi syndrome	1 (3.6)
Double cortex (*DCX* gene mutation)	1 (3.6)
Genetic^a^	3 (10.7)
Immunogenic (FIRES)	1 (3.6)
MRI negative	9 (32.1)
Number of current ASMs, median [range]	2 [1–5]
Epilepsy surgery, *n* (%)	
Performed	8 (28.6)
Engel class 1A outcome	4 (14.3)
Seizure frequency, *n* (%)	
Daily	23 (76.7)
Weekly	6 (20)
Monthly	1 (3.3)
Type of VEM analyzed, *n* (%)	
Scalp EEG	28 (93.3)
Intracranial EEG	2 (6.7)

Abbreviations: ASM, anti‐seizure medication; CdG, chapeau de gendarme; EEG, electroencephalography; FIRES, febrile infection‐related epilepsy syndrome; MRI, magnetic resonance imaging; VEM, video‐EEG monitoring.

^a^ Duplication 9q21 (*n* = 1); pathogenic gain‐of‐function KCNT1 variant (*n* = 1); pathogenic MECP2 variant (*n* = 1).

The median age in the CdG cohort was 12.3 years (range 1.3–16.8) and was higher than in the non‐CdG cohort (12.3 vs 8.9 years), although this difference did not reach statistical significance (*p* = .05). When examining age distribution descriptively, CdG was observed across all pediatric age groups but appeared more frequently in older children and adolescents (Figure 2). The youngest patient with a CdG was a 1.3‐year‐old girl (two CdG seizures), representing the only case in the 0 to <3 year group. Formal age‐based analyses refined this observation. Within the CdG cohort, CdG occurred significantly more frequently in patients aged ≥12 years compared with those younger than 12 years (*p* = .02). In contrast, a Spearman correlation analysis across the entire age range showed only a moderate, non‐significant association between age and CdG occurrence (*r* = .33, *p* = .19). Supporting a possible developmental aspect, review of earlier VEMs revealed that three patients (Patients 5, 16, and 19) developed CdG only later in the disease course, with CdG first recorded at ages 15.4, 13.7, and 12.0 years, despite prior VEMs at 10.6, 11.8, and 11.3 years showing the same seizure types.

**FIGURE 2:**
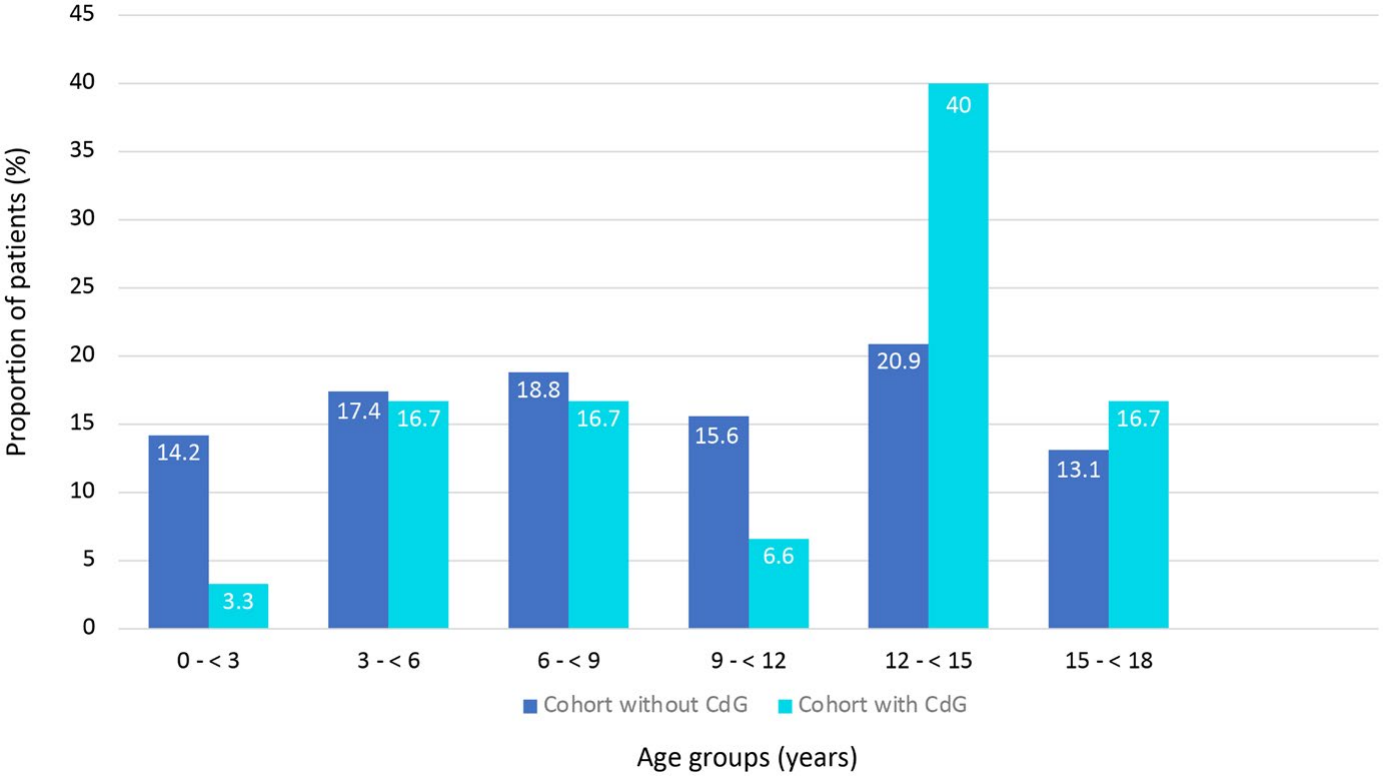
Percentage of patients with and without chapeau de gendarme (CdG) across the different age groups (based on all included video‐EEG monitoring).

### 3.3 Characteristics of seizures with CdG

CdG appeared as an early seizure sign, predominantly within the first 5 s (61.6%) and in more than 75% of seizures within the first 10 s after seizure onset. In 46.6% of CdG seizures, CdG was the first or second observable semiology. In 21.9% of seizures, it was the very first sign, usually occurring simultaneously with other manifestations such as tonic posturing, and in 6.8% did it occur as the sole initial sign. In patients with more than one CdG seizure, the latency from seizure onset to CdG onset showed little intraindividual variability. In the CdG cohort, most seizures were focal motor (75.3%). In addition, CdG seizures occurred significantly more often during sleep than seizures without CdG (*p* < .001, odds ratio [OR] = 3.07). Further characteristics of seizures with CdG are summarized in Table 2.

**TABLE 2:** Characteristics of seizures with CdG (*n* = 73).

Characteristic	Data
Latency of CdG after seizure onset^a^	
Time, s; mean [range]	8.7 [0–57]
Occurrence within 5 s, *n* (%)	45 (61.6)
Occurrence within 10 s, *n* (%)	55 (75.3)
Duration of CdG, s; mean [range]	10.1 [5–33]
Vigilance state at seizure onset, *n* (%)	
Asleep	47 (64.4)
Awake	26 (35.6)
CdG among first two semiological signs, *n* (%)	
Total number of seizures	34 (46.6)
CdG as first sign	16 (21.9)
CdG as second sign	18 (24.7)
Seizure classification, *n* (%)	
Focal motor	55 (75.3)
Focal to bilateral tonic–clonic	13 (17.8)
Focal non‐motor	4 (5.5)
Generalized tonic–clonic	1 (1.4)
Number of CdG seizures per patient having CdG, median [range]^a^	1.5 [1–10]
Percentage of CdG seizures among all analyzed seizures per patient, median [range]	20 [5.9–100]

Abbreviations: CdG, chapeau de gendarme; s, seconds.

^a^ As defined in the Section 2.

In addition to the CdG (46.6%), the most frequent other semiological signs occurring as the first or second seizure manifestation were tonic posturing (39.7%) and vegetative symptoms (12.3%). During the course of CdG seizures, HKB was present in 9.6% of all CdG seizures (*n* = 4 patients with CdG; 14.3%) and progression to BTCS was recorded in 20.5% of all CdG seizures (*n* = 5 patients with CdG; 17.9%). HKB occurred at a median of 4 s (range 0–22 s) and BTCS at a median of 7 s (range 0–38 s) after seizure onset. In all seizures with HKB, anteroposterior movements were recorded. During the movements, a pronounced emotional facial expression was observed in most patients. Compared with CdG seizures without secondary generalization, seizures evolving into FBTC were less likely to show CdG as an early semiological sign (26.6% vs 51.7%), corresponding to a later occurrence of CdG relative to seizure onset (median 7 vs 3 s), although this difference did not reach statistical significance (*p* = .25). When all EEG recordings from patients with CdG were considered—including those without recorded CdG—an even higher proportion of patients showed HKB (28.6%) and focal to bilateral tonic–clonic seizures (32.1%). Compared to patients without CdG, both BTCS (OR = 3.09) and HKB (OR = 2.46) occurred significantly more often in the CdG cohort (*p* < .001, Figure 3).

**FIGURE 3:**
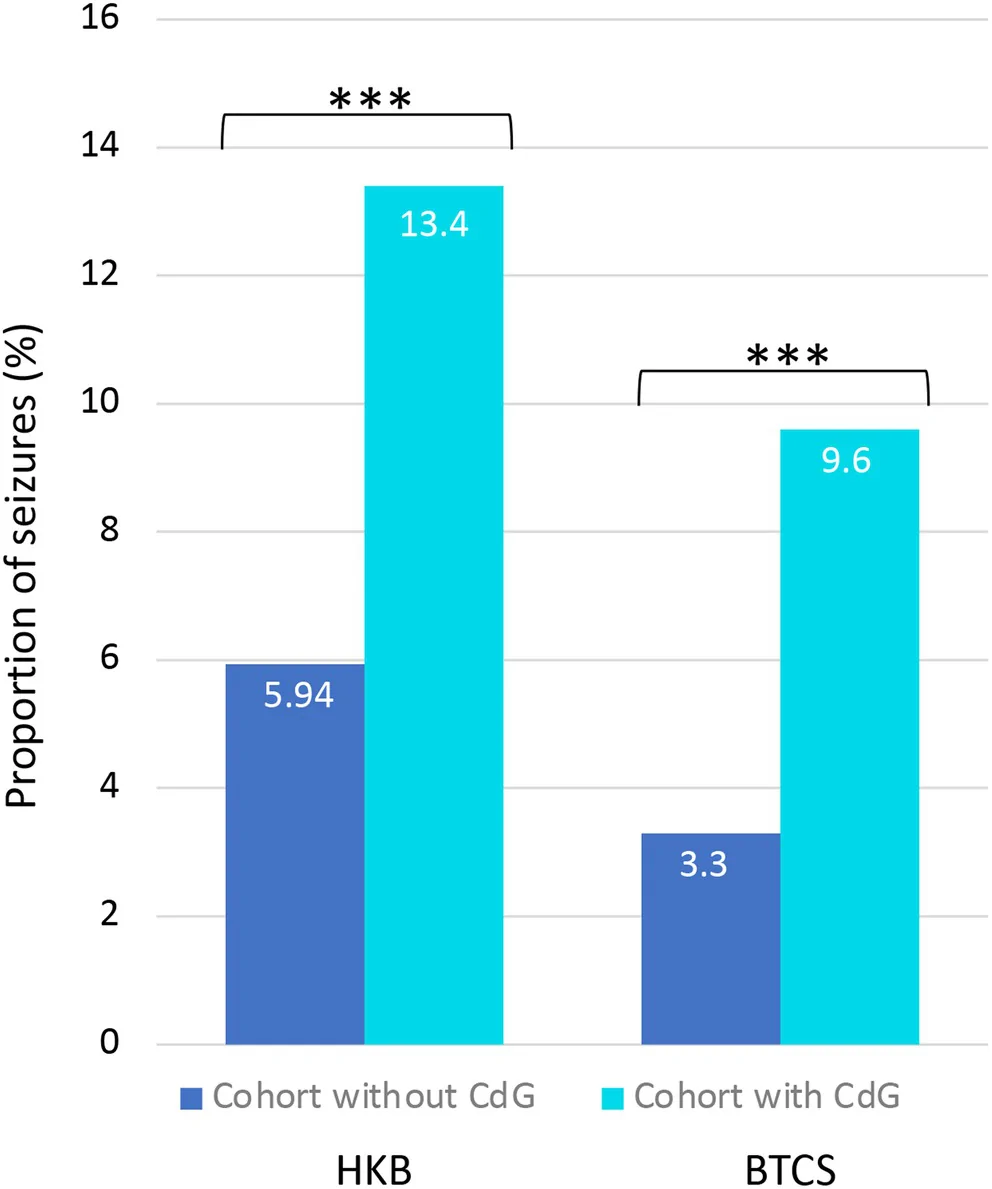
Proportion of seizures with hyperkinetic behavior (HKB) and bilateral tonic–clonic seizures (BTCS) in all analyzed seizures of the cohort with and without chapeau de gendarme (CdG) (***p < .001).

### 3.4 Localization of the first identifiable EEG ictal pattern

Although not the primary focus of this study, we also analyzed the regions in which the ictal EEG pattern first appeared in seizures with CdG. This assessment was based mainly on scalp EEG recordings (*n* = 28, 65 seizures), but data from two stereo‐EEG recordings were also included (*n* = 2; 8 seizures). Without considering the timing of CdG occurrence, the first ictal pattern was lateralized to the left hemisphere in 27 seizures (37.0%), and to the right hemisphere in 24 seizures (32.9%), whereas no clear lateralization or no detectable pattern was present in 22 seizures (30.1%). At the lobar level, the first ictal pattern was predominantly detected in the frontal lobe (57.7%, *n* = 45), followed by the temporal lobe (14.1%, *n* = 11). Insular involvement was observed in only one seizure (1.3%), based exclusively on stereo‐EEG recording, whereas in 26.9% (*n* = 21) of seizures, the pattern could not be attributed to a single lobe or was not detectable (Figure 4A). When focusing only on seizures in which a CdG appeared within 5 s of seizure onset, the proportion of frontal patterns increased (71.4%, *n* = 35), whereas the proportion of temporal (12.3%, *n* = 6) and non‐localizable patterns (16.3%, *n* = 8) decreased accordingly (Figure 4B). In one patient with epilepsy of unknown etiology and unclear classification, the scalp EEG pattern during the CdG seizure appeared generalized without further accompanying focal semiology. All other seizures with bilateral EEG patterns occurred in patients with confirmed focal epilepsy. Of the CdG seizures recorded with stereo‐EEG, one patient exhibited a single CdG seizure, in which the CdG occurred 30 s after seizure onset. In this patient, stereo‐EEG exploration suggested a widespread right‐hemispheric network, and no surgery was performed. A previous non‐invasive VEM of the same patient was also included in the analysis but showed no CdG. In the second patient, seven seizures were recorded, all with CdG within 5 s of seizure onset. Although the first ictal pattern was widespread and poorly localizable on scalp EEG, stereo‐EEG exploration localized seizure onset to the left anterior cingulate cortex (ACC). Resection of an FCD type IIa lesion resulted in sustained postoperative seizure freedom (Engel class 1A at long‐term follow‐up).

**FIGURE 4:**
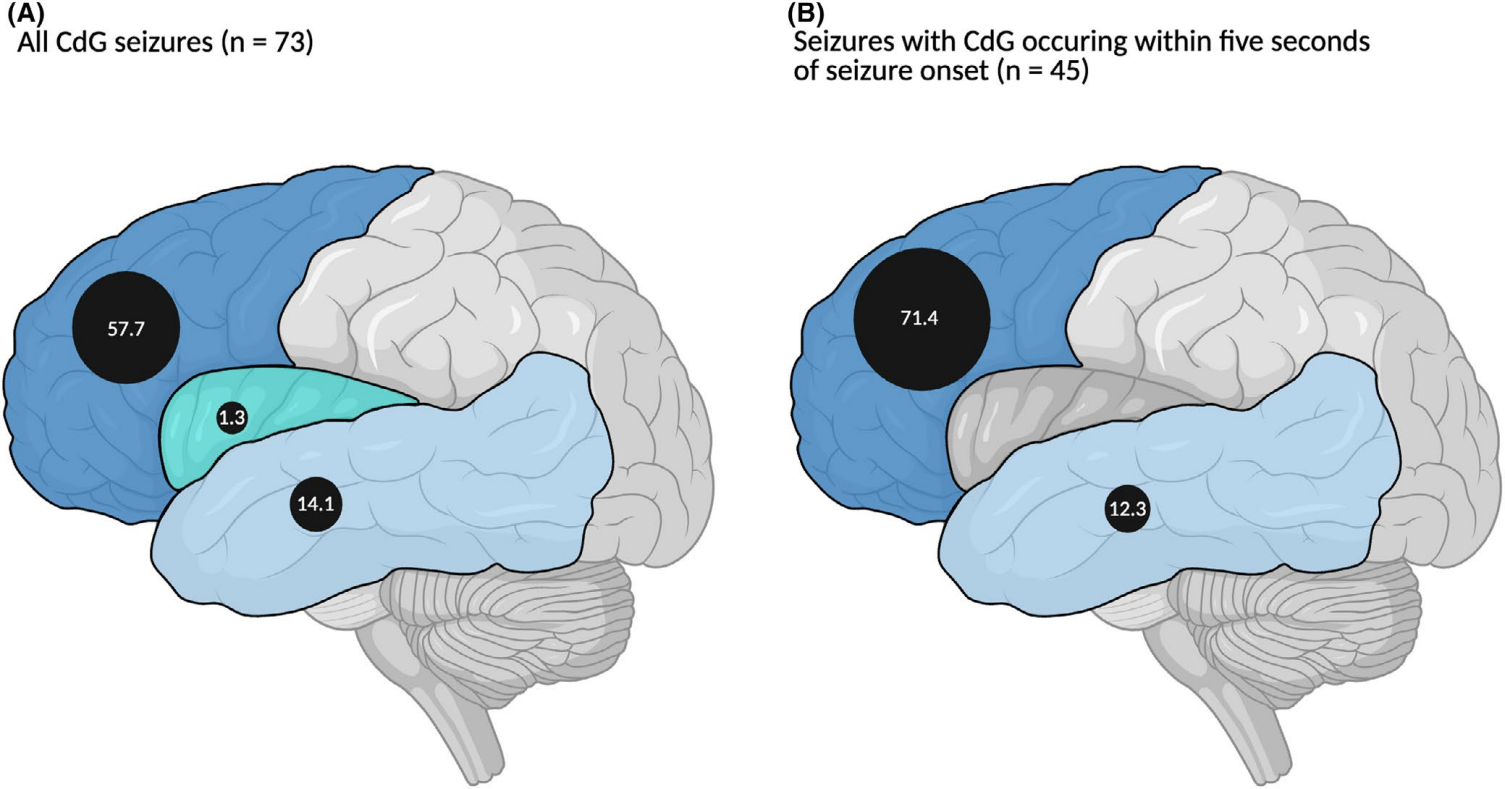
Regions of first identifiable ictal EEG patterns in seizures with chapeau de gendarme (CdG). Exemplary illustration of the brain regions where the first identifiable ictal EEG activity was recorded; for visualization purposes, all regions showing ictal activity are projected onto the left hemisphere. Circle size corresponds to the proportion of first ictal EEG patterns within each lobe, with the respective percentages indicated inside the circles. Seizures with patterns spanning two regions were included in analysis for both regions. (A) All CdG seizures (*n* = 73) from 28 scalp EEG recordings and 2 stereo‐EEG recordings. (B) Seizures with CdG occurring within 5 s of seizure onset (*n* = 45) from 15 scalp EEG recordings and 1 stereo‐EEG recording. Created in BioRender (2026), https://BioRender.com/7an4tlg.

## 4 DISCUSSION

In this study, we analyzed the occurrence and characteristics of CdG in a large pediatric cohort covering the full age range from infancy to adolescence, using systematically reviewed video‐EEG data. CdG was identified in 10.6% of all patients, occurring from as early as 1.3 years of age up to 16.8 years, with a tendency to appear more frequently in older children. It typically emerged within the first seconds of seizure onset, often co‐occurring with HKB and BTCS in the seizure course, and predominantly emerging during sleep. CdG was frequently associated with structural etiologies, most commonly focal cortical dysplasia.

In contrast to earlier reports that described CdG as an extremely rare finding in pediatric epilepsy,^14^ our results demonstrate that CdG occurred in a notable proportion of patients. Across the entire cohort, 73 CdG seizures were identified in 10.6% of patients. The true frequency is likely even higher, as only up to 10 seizures per type were analyzed for each patient, and in some cases partial masking (e.g., the mouth not visible after a few seconds) may have led to underdetection. The two previous studies on CdG in adults and children were conducted in highly selected surgical cohorts with refractory epilepsy and small sample sizes (10–11 patients). These studies focused primarily on the localizing aspects of CdG and did not provide data on its overall frequency in pediatric epilepsy.^9^, ^11^ In broader studies assessing semiological frequencies in children, CdG has not been reported as a category and may have been overlooked, as it is a subtle sign that can easily be masked by more prominent seizure manifestations.^19^ Another challenge in comparing CdG frequencies across studies lies in its inconsistent definition. At the time of data acquisition and analysis in our study, standardized and widely accepted criteria for CdG were still limited,^10^ which is why duration was used as a key operational criterion. The reported duration thresholds vary considerably: the International League Against Epilepsy suggests a minimum duration of more than 3 s,^8^ whereas Souriti et al. required at least 5 s,^9^ and one case report even classified events lasting as little as 2.2 s as CdG.^20^ However, duration alone is unlikely to fully capture the complexity of this semiology. The clinical picture—particularly the global facial expression—may provide important insights into the underlying epileptogenic network. Recent studies hypothesize that mire emotionally expressive forms of CdG may be associated with mesial frontal regions, including the ACC, whereas less expressive forms may reflect involvement of motor or opercular networks.^21^ We therefore propose that CdG should be characterized primarily by its clinical features, with duration serving as a supportive criterion to distinguish it from brief or nonspecific facial movements.

The age at which CdG first appears as a semiological sign and potential age‐related differences in its occurrence have not been systematically analyzed to date. Previous reports were based mainly on small surgical cohorts consisting predominantly of adults or on single case studies, focusing on the localizing value of CdG rather than its epidemiology.^9^, ^11^ The youngest patient reported with CdG seizures was a 14‐month‐old girl,^16^ whereas most other pediatric cases described in the literature were older children and adolescents 6–18 years of age.^9^, ^11^, ^14^, ^15^, ^22^ In our cohort, CdG was observed across the pediatric age range from 1.3 to 16.8 years. CdG occurred more frequently in older children, although formal correlation analysis did not reach statistical significance. This likely reflects the unequal age distribution in our sample, with younger children—particularly those aged 0 to <3 years—being underrepresented. Notably, three patients developed CdG only later in the disease course despite earlier VEMs showing the same seizure types without CdG. Together, these findings suggest that the emergence of CdG may be influenced by brain maturation and the evolving expression of complex motor semiologies. However, future studies including larger numbers of infants and younger children are required to determine whether the observed age‐related pattern reflects a true developmental phenomenon or is attributable primarily to sampling bias.

Consistent with previous studies and case reports,^9^, ^14^ CdG appeared as an early and short‐latency seizure sign in the majority of cases: more than 60% of CdG manifestations occurred within 5 s of seizure onset, and about 75% within 10 s. Furthermore, it was the first seizure semiology in 21.9% of CdG seizures, with additional semiologies partly arising only later in the seizure course. These findings, together with two case reports describing CdG as the only ictal manifestation throughout the entire seizure,^15^, ^19^ underscore its diagnostic relevance and potential role in seizure recognition, epileptogenic zone identification, and localization of early propagation networks. Of interest, CdG was significantly more frequent during sleep, suggesting that state‐dependent modulation of cortical–subcortical connectivity might facilitate its occurrence. Sleep is known to promote synchronization in frontal and limbic circuits. These circuits have been implicated in the generation of CdG in invasive EEG studies.^9^, ^11^, ^15^, ^20^, ^22^

Previous studies have described the co‐occurrence of HKB with CdG but reported frequencies vary widely.^9^, ^11^, ^14^ In our cohort, HKB was observed in 14.3% of all patients with CdG and occurred significantly more often in these patients compared with those without CdG seizures. HKB is known to appear more often with advancing age,^4^, ^5^, ^23^ which may partly account for this association, given that CdG was more common in older children and our cohort also included a relatively high proportion of adolescents. Previous CdG studies have not described the types of HKB movements or their co‐occurring semiologies. The observed anteroposterior movements and emotional facial expressions resemble the type 1 hyperkinetic seizure presented in the study by Rheims et al., who differentiated two types of HKB in patients with seizures involving the frontal cortex. In their study, type 1 was significantly associated with a seizure‐onset zone in the ventromesial cortex.^18^ A more recent study using a multidimensional assessment of HKB proposed three clusters of hyperkinetic seizures, each associated with a neural network involving different brain regions. Emotionality, together with vocalization, represents the principal feature of Cluster 3 and is a common feature of our finding. This cluster has been linked to ventromesial prefrontal and temporal regions.^24^ However, because our study was based on scalp EEG recordings and additional cluster characteristics, such as motor asymmetry or temporal dynamics, were not analyzed, the involvement of these networks cannot be confirmed. Further studies are required to determine the role of specific cortical regions within the networks underlying seizures with CdG accompanied by HKB. Seizures with CdG also progressed to BTCS in a subset of patients (17.9% of patients with CdG), and were significantly more frequent in the CdG cohort compared to the non‐CdG cohort. This co‐occurrence has not been reported in previous studies with pediatric patients, which included only small or highly selected samples. As BTCS themselves become more common with advancing age,^4^ this association may reflect both a developmental phenomenon and cohort composition, warranting further investigation. However, given the small total number of patients in whom this was observed, the finding should be interpreted with caution.

Although not the main focus of this study, we also analyzed the regions where the first identifiable ictal EEG activity occurred in CdG seizures: Patterns most often involved the frontal lobe, followed by the temporal lobe. Previous intracranial studies have linked CdG to seizures involving frontal, premotor, fronto‐limbic, insular, and temporal regions, suggesting that distributed networks rather than a single anatomic structure underlie this semiology.^9^, ^10^, ^11^, ^14^, ^16^, ^20^, ^25^, ^26^ In particular, studies using intracranial recordings have associated CdG with activation of premotor and medial frontal networks including the ACC.^8^, ^9^, ^10^, ^11^, ^19^, ^20^, ^21^ However, as almost all recordings in our study were scalp EEG studies, the involvement of deeper structures such as the insula and the specific role of individual regions, including the ACC, cannot be conclusively determined based on our data. Nevertheless, the frequent co‐occurrence of HKB in our study—which has been linked previously to frontal and cingulate regions^18^, ^24^, ^27^, ^28^—is consistent with the involvement of these networks in CdG generation and with the concept of a shared network underlying seizures with CdG and HKB, as proposed in previous studies.^9^, ^11^, ^20^ CDG is considered by some authors to be a primarily motor sign,^26^ whereas others consider it to be a sign linked to an emotional–behavioral system.^9^, ^11^ Further studies are required to better define the role of these structures in the generation of CdG. Consistent with previous findings,^9^, ^10^ CdG was not a lateralizing sign in our study. CdG has been described exclusively in the literature as a purely focal symptom.^10^ In line with this, focal epilepsy was significantly more frequent in our cohort among patients with CdG than in those without (*p* = .009). In one patient with epilepsy of unknown etiology and unclassified focal or generalized onset, a generalized appearing scalp EEG pattern was observed during a CdG seizure. Given the unclear epilepsy classification in this case, this finding does not allow conclusions regarding a truly generalized origin of CdG. Although focal semiologies can occasionally occur in generalized seizures due to transient or asymmetric cortical network activation,^29^ whether this also applies to CdG remains uncertain and cannot be determined from our data.

Several limitations of this study should be acknowledged. First, as a retrospective, single‐center study conducted at a tertiary epilepsy center with a focus on presurgical evaluation, our cohort is subject to selection bias toward patients with drug‐resistant focal epilepsies, whereas generalized epilepsies were likely underrepresented. Consequently, the findings on CdG frequency and age distribution may not fully reflect the broader pediatric epilepsy population. Second, most data were derived from scalp EEG recordings, which are limited in spatial resolution and sensitivity—particularly for activity arising from deep or mesial structures such as the insula or ACC. The apparent predominance of frontal patterns may therefore, at least in part, reflect methodological constraints.

Third, semiological assessment inevitably involves some subjectivity, especially for subtle signs such as CdG, which may be obscured by other motor phenomena or restricted camera views. Despite systematic video review and standardized evaluation, underrecognition cannot be entirely excluded. Finally, the uneven age distribution of our cohort, with fewer patients in early childhood, limits the interpretation of developmental aspects of CdG occurrence. Nevertheless, our study provides the most comprehensive characterization of CdG seizures in pediatric epilepsy to date. By defining their frequency, clinical context, and EEG correlates across a broad age range, it establishes an essential foundation for future studies focused on the precise localization and network mechanisms underlying this distinctive semiology.

## 5 CONCLUSION

CdG is a subtle yet clinically relevant seizure semiology in children, typically appearing as an early sign in focal seizures. By defining its frequency and clinical characteristics, our study provides an essential framework for recognizing CdG in pediatric video‐EEG and for future investigations into its developmental and network mechanisms.

## AUTHOR CONTRIBUTIONS

**Kerstin Alexandra Klotz**: Conceptualization (lead); formal analysis (lead); investigation (lead); methodology (lead); project administration (lead); resources (lead); visualization (supporting); and writing—original draft (lead). **Hanna Barbara Brinkmann**: Conceptualization (supporting); formal analysis (lead); investigation (lead); methodology (lead); project administration (supporting); visualization (lead); and writing—original draft (lead). **Lea Nakamura**: Conceptualization (lead); resources (supporting); and writing—review & editing (equal). **Victoria San Antonio‐Arce**: Resources (supporting) and writing—review & editing (equal). **Jan Schönberger**: Writing—review & editing (equal). **Julia Jacobs**: Writing—review & editing (equal).

## FUNDING INFORMATION

This research did not receive any specific grant from funding agencies in the public, commercial, or not‐for‐profit sectors.

## CONFLICT OF INTEREST STATEMENT

The authors declare no conflicts of interest.

## CONSENT

For this retrospective study, the requirement for written informed consent for the use of clinical data was waived by the local ethics committee. Written informed consent for publication of clinical images was obtained.

## ETHICAL PUBLICATION STATEMENT

This study was approved by the ethics committee of the University Medical Center Freiburg (file 45/19). We confirm that we have read the Journal's position on issues involved in ethical publication and affirm that this report is consistent with those guidelines.

## Supporting information


Table S1.

## Data Availability

The data that support the findings of this study are available on reasonable request from the corresponding author. The data are not publicly available due to privacy or ethical restrictions.
