# Supplementary material for: Characterizing chapeau de gendarme in pediatric epilepsy through systematic video‐EEG evaluation
Source: Epilepsia. 2026 Jul 23;67(10):5301–11. doi: 10.1002/epi.70396 (PMC13675766; doi:10.1002/epi.70396)
Supplement: Supplementary file 1 [file EPI-67-5301-s001.docx]

**Table S1: Clinical characteristics of individual patients and their CdG characteristics in the Video-EEG Monitoring**

| **No. Pat.\|**  **Gender** | **Type of epilepsy\|Etiology** | **Number of CdG seizures** | **Age at first CdG (y)** | **Mean occurrence after seizure onset (s)** | **add. HKB** | **add. BTCS** | **Type of EEG** | **Region first seizure pattern** | **Surgery\|Outcome (Engel classification)** |
| --- | --- | --- | --- | --- | --- | --- | --- | --- | --- |
| 1\|M | Structural\|Ganglioglioma Temporomesial L | 1 | 8.8 | 31 | - | - | S | T L | TL resection + AHE\|1A |
| 2\|M | Structural\|Defect after resection and radiotherapy of a pilocytic astrocytoma | 10 | 14.1 | 1.4 | - | - | S | FT R or FC R | - |
| 3\|W | Unknown | 1 | 12.5 | 9 | - | - | S | T L | - |
| 4\|M | Structural\|Pleomorphic xanthoastrocytoma Temporo-basal/posterior R | 2 | 14.7 | 40.5 | - | - | S | bi. | Extended lesionectomy\|MD |
| 5\|M | Unknown | 1 | 15.4 | 30 | - | - | I | anterior Insula and mesial F | - |
| 6\|W | Unknown | 1 | 15.1 | 6 | + | - | S | bi. | - |
| 7\|W | Structural-genetic\|Double cortex with  mutation in the DCX-gene | 1 | 4.2 | 57 | - | - | S | T R | - |
| 8\|M | Structural-genetic\|TSC | 1 | 5.2 | 13 | - | - | S | F L | - |
| 9\|W | Unknown | 5 | 8.2 | 2.8 | - | - | S | T L or F L | - |
| 10\|M | Genetic\|Duplication 9q21 | 2 | 14.7 | 12.5 | - | - | S | F bi. | - |
| 11\|W | Unknown | 4 | 10.7 | 12.5 | - | - | S | FP R or bi. or unknown | - |
| 12\|W | Structural\|FCD type IIb Temporolateral R | 2 | 7.1 | 0.5 | - | - | S | FT R or F R | Extended lesionectomy\|1A |
| 13\|W | Genetic\|Mutation in the KCNT1 gene | 1 | 5.1 | 4 | + | - | S | F R | - |
| 14\|M | Structural\|Complex bilateral brain malformation | 5 | 12.1 | 1.6 | - | + | S | FC L or FT bi. or bi. | - |
| 15\|W | Unknown | 1 | 16.8 | 38 | - | + | S | gen. | - |
| 16\|W | Genetic\| Mutation in the MECP2-Gen | 1 | 13.7 | 1 | - | - | S | bi. | - |
| 17\|W | Structural\|FCD type IIa Frontal L | 4 | 8.7 | 8 | + | + | S | F L | Extended lesionectomy\|1A |
| 18\|M | Structural\|FCD type IIa ant. cingulate L | 7 | 14.1 | 1 | - | - | I | F L | Extended lesionectomy\|1A |
| 19\|M | Structural\|Cortical malformation Temporal R | 1 | 12 | 33 | - | + | S | T R | TL resection + AHE R \|1C |
| 20\|W | Unknown | 1 | 12.4 | 0 | - | - | S | F R | - |
| 21\|W | Structural-genetic\|Aicardi syndrome | 2 | 1.3 | 1.5 | - | - | S | unknown | - |
| 22\|W | Structural\|FCD type IIa Frontal L | 4 | 14.7 | 5.8 | + | - | S | F L | Extended lesionectomy \|1B |
| 23\|W | Unknown | 5 | 3.4 | 8.4 | - | - | S | bi. or unknown | - |
| 24\|M | Immunogenic\|FIRES | 1 | 6.4 | 5 | - | + | S | T L | - |
| 25\|M | Structural-genetic\|TSC | 4 | 5.7 | 8.8 | - | - | S | unknown | - |
| 26\|M | Structural\|Oligodendroglioma | 1 | 16 | 0 | - | - | S | bi. | Lesionectomy\|3A |
| 27\|W | Structural\|FCD Frontal R | 1 | 12.7 | 3 | - | - | S | unknown | - |
| 28\|M | Unknown | 3 | 15 | 22.7 | - | - | S | F R | - |

Abbreviations: add. = additional, ant. = anterior, AHE = amygdalohippocampectomy, BTCS = bilateral tonic-clonic seizure, bi = bilateral, F = frontal, FC = fronto-central, FCD = Focal cortical dysplasia, FIRES = febrile infection-related epilepsy syndrome, FP = fronto-polar, FT = fronto-temporal, gen = generalized, HKB = Hyperkinetic behaviour, I = intracranial EEG, L = left, MD = missing data, mv = mean value, No. = number, Pat. = patient, R = right, s = seconds, S = scalp EEG, T = temporal, TL = temporal lobe, TSC = tuberous sclerosis, y = years, + = yes, - = no
